# Rescue therapy for patients with anti-PD-1-refractory Merkel cell carcinoma: a multicenter, retrospective case series

**DOI:** 10.1186/s40425-019-0661-6

**Published:** 2019-07-08

**Authors:** Jaclyn LoPiccolo, Megan D. Schollenberger, Sumia Dakhil, Samuel Rosner, Osama Ali, William H. Sharfman, Ann W. Silk, Shailender Bhatia, Evan J. Lipson

**Affiliations:** 1 Department of Medicine, Johns Hopkins University School of Medicine, Baltimore, MD USA; 2 Department of Oncology, Sidney Kimmel Comprehensive Cancer Center, and Bloomberg-Kimmel Institute for Cancer Immunotherapy, Johns Hopkins University School of Medicine, 1550 Orleans Street, Room 507, Baltimore, MD 21287 USA; 3 Department of Medicine/Medical Oncology, University of Washington and Fred Hutchinson Cancer Research Center, Seattle, Washington USA; 4 Department of Medicine, Johns Hopkins Bayview Medical Center, Baltimore, MD USA; 5 Department of Radiology, Johns Hopkins University School of Medicine, Baltimore, MD USA; 6 Rutgers Cancer Institute of New Jersey, New Brunswick, NJ USA

**Keywords:** Merkel cell carcinoma, Anti-PD-1-refractory, Progression, Immune checkpoint blockers

## Abstract

Merkel cell carcinoma (MCC) is a rare but clinically aggressive cancer with a high mortality rate. In recent years, antibodies blocking the interactions among PD-1 and its ligands have generated durable tumor regressions in patients with advanced MCC. However, there is a paucity of data regarding effective therapy for patients whose disease is refractory to PD-1 pathway blockade. This retrospective case series describes a heterogeneous group of patients treated with additional immune checkpoint blocking therapy after MCC progression through anti-PD-1. Among 13 patients treated with anti-CTLA-4, alone or in combination with anti-PD-1, objective responses were seen in 4 (31%). Additionally, one patient with MCC refractory to anti-PD-1 and anti-CTLA-4 experienced tumor regression with anti-PD-L1. Our report – the largest case series to date describing this patient population – provides evidence that sequentially-administered salvage immune checkpoint blocking therapy can potentially activate anti-tumor immunity in patients with advanced anti-PD-1-refractory MCC and provides a strong rationale for formally testing these agents in multicenter clinical trials. Additionally, to the best of our knowledge, our report is the first to demonstrate possible anti-tumor activity of second-line treatment with a PD-L1 antibody in a patient with anti-PD-1-refractory disease.

**Electronic supplementary material:**

The online version of this article (10.1186/s40425-019-0661-6) contains supplementary material, which is available to authorized users.

## Introduction

Immune checkpoint blocking therapy has transformed the treatment landscape for patients with numerous tumor types, including those with Merkel cell carcinoma (MCC) [1]. Over the last few years, agents blocking the immunoregulatory pathway comprised of PD-1 and its ligands have demonstrated anti-tumor activity in ~ 30–60% of patients, as well as improvements in progression-free and overall survival compared to historical data from patients receiving cytotoxic chemotherapy [2–6]. Reflecting this, the National Comprehensive Cancer Network’s 2019 guidelines include avelumab (anti-PD-L1), pembrolizumab (anti-PD-1) and nivolumab (anti-PD-1) as preferred therapies for patients with advanced MCC [7]. However, despite these advancements, a substantial portion of patients require “rescue” therapy in the setting of MCC refractory to PD-(L)1 monotherapy. Here, we report the largest case series to date of patients with advanced MCC who received additional immune checkpoint blocking therapies following disease progression on or shortly after treatment with anti-PD-1. We provide evidence that anti-CTLA-4, with or without radiation therapy, can activate synergistic anti-tumor immunity in this patient population. In addition, to the best of our knowledge, our report is the first to demonstrate the efficacy of second-line treatment with a PD-L1 antibody in a patient with anti-PD-1-refractory disease.

## Materials and methods

We reviewed available records of patients at two large academic medical centers (Sidney Kimmel Comprehensive Cancer Center at Johns Hopkins and the University of Washington / Fred Hutchinson Cancer Research Center) who were treated with alternative immune checkpoint blocking therapy after MCC progression during or shortly after receiving anti-PD-1/PD-L1. Demographic patient data and information about disease status were collected via chart review. Radiographic outcomes were measured using RECIST v1.1 and immune-related response criteria [8, 9].

## Results

Among 13 patients treated with anti-CTLA-4, objective responses were seen in 4 (31%). Notably, one patient with MCC refractory to anti-PD-1 and anti-CTLA-4 experienced tumor regression after anti-PD-L1 + radiotherapy. These cases are described below.

### Case 1

**Fig. 1:**
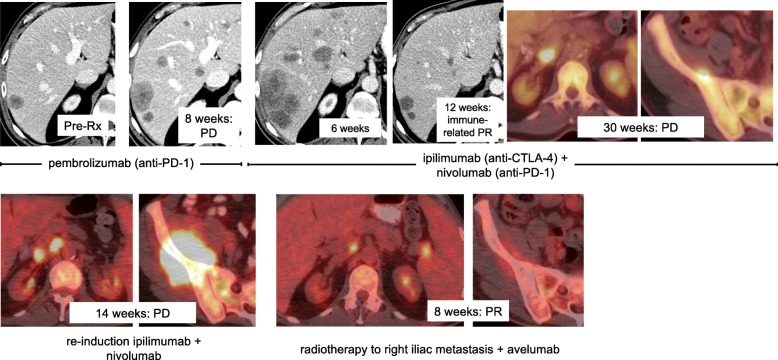
Representative computed tomography (CT) and positron emission tomography (PET)/CT images from a 67-year-old man with advanced Merkel cell carcinoma. He experienced progressive disease after receiving pembrolizumab (two upper left panels), then an immune-related partial response to ipilimumab + nivolumab lasting 30 weeks (four upper right panels). Re-induction ipilimumab + nivolumab administered at the time of disease progression was ineffective in regaining disease control (two lower left panels). However, administration of avelumab and radiotherapy to a right iliac metastasis resulted in a partial response (RECIST v1.1) at 8 weeks. PR lasted 12 months

A 67-year-old man with Lynch syndrome (*MSH6* mutation) and polycythemia vera presented with histologically-proven MCC (unknown Merkel cell polyomavirus (MCPyV) status) metastatic to the liver. He received first-line therapy with pembrolizumab for 2 months with progressive disease (PD) as his best response. (Fig. 1) He was then treated with four cycles of ipilimumab (anti-CTLA-4, 3 mg/kg) + nivolumab (anti-PD-1, 1 mg/kg) every 3 weeks × 4 and experienced a partial response per immune-related response criteria, which lasted 30 weeks before his disease progressed. Ipilimumab + nivolumab was administered again but resulted in PD at 14 weeks. The patient then received avelumab (anti-PD-L1) 10 mg/kg every 2 weeks plus radiotherapy (3D conformal radiation therapy, 2500 centigray) to a right iliac mass, which resulted in a partial response (PR) per RECIST v1.1. Marked regression was also noted in the irradiated tumor (Fig. 1) and the patient’s Eastern Cooperative Oncology Group (ECOG) performance status improved from 2 to 0. PR lasted 12 months.

### Case 2

**Fig. 2:**
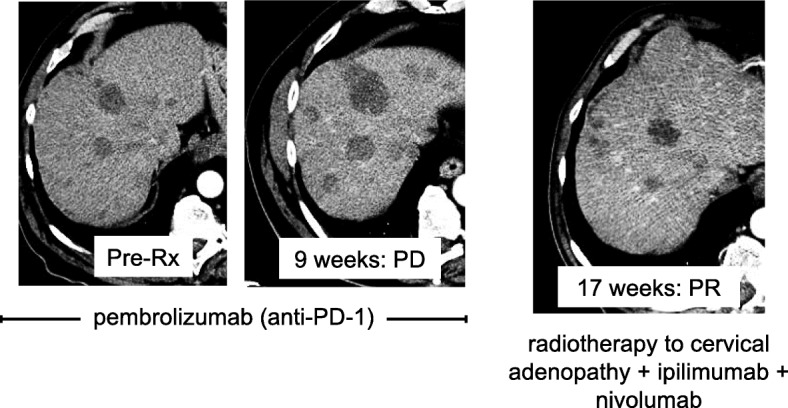
Representative CT images from a 79-year-old man with advanced Merkel cell carcinoma. He experienced progressive disease after receiving pembrolizumab (two left panels). He then received ipilimumab + nivolumab + radiotherapy to cervical adenopathy, to which he developed a partial response (RECIST v1.1) 17 weeks into combinatorial therapy (right panel). PR was ongoing at 8 months when the patient died from complications related to encephalopathy, likely immune-mediated (i.e., a toxicity related to immune checkpoint inhibitor therapy)

A 79-year-old man presented with cervical lymphadenopathy and liver metastases from a primary MCC on the right cheek (unknown MCPyV status). He was treated with pembrolizumab and experienced PD at 9 weeks. (Fig. 2) He then received ipilimumab (3 mg/kg) + nivolumab (1 mg/kg) every 3 weeks × 4 followed by nivolumab monotherapy (3 mg/kg) every 2 weeks, along with intensity-modulated radiation therapy (IMRT, 4000 cGy) to cervical tumors. He experienced a PR per RECIST v1.1 (Fig. 2) at 17 weeks. In the setting of an ongoing PR at 8 months, the patient developed profound fatigue and altered mental status of unclear etiology, possibly a result of an immune-mediated adverse reaction (e.g., encephalitis) associated with immune checkpoint blocking therapy. The patient declined further workup and died 2 months later from complications related to encephalopathy.

### Case 3

A 59-year-old man presented with symptomatic, widely metastatic MCPyV-positive MCC that progressed through > 5 therapeutic regimens, including surgery, radiotherapy (RT), cytotoxic chemotherapy, intra-tumoral (IT) interferon-beta, IT interleukin (IL)-12, somatostatin analogues, and adoptive T cell therapy (ACT) with MCPyV-specific T cells plus pembrolizumab. The patient had received 3 doses of pembrolizumab (1 pre-ACT and 2 post-ACT) and, despite persistence of infused T cells in the peripheral blood, the patient’s best response was PD. After considering the possibility of best supportive care (i.e., hospice), the patient opted to receive one dose of ipilimumab (50 mg; 0.5 mg/kg). Two days after ipilimumab infusion, the patient reported flu-like symptoms reminiscent of cytokine release. Over the next few weeks, he experienced a dramatic clinical improvement; a restaging evaluation at 6 weeks demonstrated a PR with > 90% tumor regression. Given this remarkable response, the patient started receiving pembrolizumab plus low-dose (50 mg) ipilimumab infusions. He maintained a PR over the next 18 months, after which he developed rapid disease progression and died. Tumor biopsy at the time of progression revealed downregulation of MHC-I expression on MCC tumor cells as a possible mechanism of acquired resistance to immune checkpoint inhibitor therapy [10]. Given this patient’s limited exposure to pembrolizumab (3 doses) and persistence of MCPyV-specific T cells in the peripheral blood prior to administration of low-dose ipilimumab, it is difficult to tease apart the individual contributions of each therapy. However, the close temporal relationship between initiation of ipilimumab and the patient’s dramatic clinical improvement supports a therapeutic synergy between the 3 agents.

### Case 4

A 71-year-old man presented with asymptomatic, MCPyV-positive MCC that progressed through > 5 therapeutic regimens, including surgery, RT, cytotoxic chemotherapy, IT IL-12, an IT toll-like receptor (TLR)-4 agonist, somatostatin analogues, and nivolumab. The patient had been receiving nivolumab for > 2 years with a complete response (CR), but 26 months after initiation of nivolumab he developed PD with asymptomatic portacaval and left iliac lymphadenopathy. Ipilimumab (1 mg/kg every 6 weeks) was added to nivolumab (3 mg/kg every 2 weeks), mirroring the regimen used in an ongoing clinical trial (ClinicalTrials.gov Identifier: NCT02488759). The patient again experienced a CR that lasted 10 months after starting ipilimumab.

## Discussion

MCC is a rare and clinically aggressive tumor with a rising incidence and a high mortality rate that is responsible for ~ 3000 deaths each year in the United States [11, 12]. Approximately 80% of MCC cases are associated with the Merkel cell polyomavirus (MCPyV) which inserts into the Merkel cell genome, eliciting an immune response [13]. The remaining ~ 20% of MCCs (i.e., MCPyV-negative tumors) occur more commonly in geographic areas with high levels of sun exposure (e.g., Australia), and have higher genetic mutational burdens than their MPCyV-positive counterparts, likely as a result of cumulative exposure to ultraviolet light [14]. MCC is highly immunogenic, though it often evades immune-mediated eradication, especially in immunosuppressed individuals (e.g., patients with chronic lymphocytic leukemia or HIV, or solid organ transplant recipients) [15]. Over half of MCC tumors express immune checkpoint molecules, including PD-1 and PD-L1 [16]. Over the last few years, these characteristics have provided a rationale for testing PD-1-pathway blockers in patients with advanced MCC. In some cases, anti-tumor responses to these therapies have been profound and durable. However, there is a paucity of data regarding effective therapy for patients whose disease is refractory to anti-PD-1/L1. The current report describes the clinical characteristics and treatment outcomes of 13 patients with advanced MCC, whose pre-treatment and treatment regimens are acknowledged to be quite heterogeneous, but who nonetheless experienced PD after administration of anti-PD-1 and were treated with additional immune checkpoint inhibitor therapy. Additional file 1: Table S1 provides an overview of all 13 patients. The cases described herein suggest a potential role for “rescue” immune checkpoint blockade in this patient population.

Successful activation of anti-tumor immunity in our patient cohort by adding anti-CTLA-4 is, perhaps, not surprising, but remarkable nevertheless for this population with few effective immunotherapy options. CTLA-4 and PD-1 contribute to related but non-overlapping immunoregulatory pathways, and in patients with other tumor types, combinatorial therapy has triggered cancer regressions after progression on anti-PD-1 therapy [17]. Anecdotally, CTLA-4 inhibition with ipilimumab has demonstrated anti-tumor activity in a small series of MCC cases [18], and is currently being investigated in combination with nivolumab in patients with advanced MCC or virus-associated cancer (ClinicalTrials.gov Identifier: NCT02488759).

To the best of our knowledge, our report is the first to describe what may have been non-identical, synergistic anti-tumor activity after sequential administration of antibodies blocking PD-1 and PD-L1 in an individual patient (case #1). Although the possibility of a delayed response to re-induction ipilimumab + nivolumab must be considered, the close temporal association between administration of avelumab + radiotherapy and the patient’s clinical and radiographic improvement supports the development of anti-tumor immunity brought about by blockade of anti-PD-L1. While it is true that PD-1 and PD-L1 are constituents of a shared immunoregulatory pathway, our observations suggest that patients who experience disease progression after blockade of one may benefit from blockade of the other. Mechanistically, PD-1 interacts with PD-L1 and PD-L2, but PD-L1 also interacts with B7.1 [19]. These interactions potentially introduce non-redundant – and possibly synergistic – mechanisms of immunoactivation. Additionally, nivolumab and pembrolizumab – both IgG4 isotype antibodies – lack antibody-dependent cytotoxicity (ADCC) [20]. In contrast, avelumab is an IgG1 monoclonal antibody with a native Fc region which triggers ADCC in addition to immune checkpoint inhibition [21–23].

Finally, some patients in our series (e.g., cases 1 and 2, described above) received radiotherapy in addition to an immune checkpoint blocker. Though determination of radiation-induced sensitization to immunotherapy (e.g., by increasing inflammatory chemokine secretion of irradiated tumor cells) remains elusive, the possibility of an abscopal effect (regression of a distant metastasis after application of local radiotherapy) should be considered [24]. MCC is known to be exquisitely sensitive to radiation therapy, and it is not known whether the radiation therapy works as a debulking agent, an immunologic adjuvant, or both.

Taken together, the outcomes described in our report demonstrate that combinatorial sequential immune checkpoint blocking agents can activate anti-tumor immunity in patients for whom anti-PD-1/L1 alone is insufficient. Of particular interest are the tumor regressions seen after sequencing PD-1- and PD-L1-blocking antibodies, which appear to potentially have non-redundant anti-cancer properties in an individual patient. Our findings require further exploration among larger cohorts of patients.

## Additional file

Additional file 1: **Table S1.** Therapies administered and corresponding disease outcomes for patients with advanced Merkel cell carcinoma refractory to anti-PD-1 or anti-PD-L1. (CLL, chronic lymphocytic leukemia; CR, complete response; PR, partial response; irPR, immune-related partial response; MCPyV, Merkel cell polyomavirus; PD, progressive disease; RT, radiotherapy). (DOCX 19 kb)

## Data Availability

The datasets used and/or analyzed during the current study are available from the corresponding author on reasonable request.

## Abbreviations

ACT: Adoptive T cell therapy
ADCC: Antibody-dependent cytotoxicity
CR: Complete response
CT: Computed tomography
ECOG: Eastern Cooperative Oncology Group
IL: Interleukin
IMRT: Intensity-modulated radiation therapy
IT: Intra-tumoral
MCC: Merkel cell carcinoma
MCPyV: Merkel cell polyomavirus
PD: Progressive disease
PD-1: programmed death 1
PD-L1: Programmed death ligand-1
PD-L2: Programmed death ligand-2
PET: Positron emission tomography
PR: Partial response
RECIST: Response evaluation criteria in solid tumors
RT: Radiotherapy
TLR: Toll-like receptor
