# Supplementary material for: Rescue therapy for patients with anti-PD-1-refractory Merkel cell carcinoma: a multicenter, retrospective case series
Source: J Immunother Cancer. 2019 Jul 8;7:170. doi: 10.1186/s40425-019-0661-6 (PMC6615256; doi:10.1186/s40425-019-0661-6)
Supplement: Supplementary file 1 — Table S1. Therapies administered and corresponding disease outcomes for patients with advanced Merkel cell carcinoma refractory to anti-PD-1 or anti-PD-L1. (CLL, chronic lymphocytic leukemia; CR, complete response; PR, partial response; irPR, immune-related partial response; MCPyV, Merkel cell polyomavirus; PD, progressive disease; RT, radiotherapy). (DOCX 19 kb) [file 40425_2019_661_MOESM1_ESM.docx]

**Additional file 1: Table S1**

| Case # | Patient age, sex, MCPyV status | Therapy #1 | Response(s) | Therapy #2 | Response(s) | Therapy #3 | Response(s) | Therapy #4 | Response(s) | Comments |
| --- | --- | --- | --- | --- | --- | --- | --- | --- | --- | --- |
| 1 | 67 M,  unknown | Pembrolizumab 2mg/kg q3wks | PD at 2 months | Ipilimumab 3mg/kg + nivolumab 1mg/kg q3wks x 4 | irPR at 9 wks, PD at 30 wks | Ipilimumab 3mg/kg + nivolumab 1mg/kg q3wks x 4 | PD at 14 wks | avelumab 10 mg/kg q2wks + RT | PR at 8 wks, PD at 12 mos | Case 1 described in manuscript |
| 2 | 79 M,  unknown | Pembrolizumab 2mg/kg q3wks | PD at 9 wks | RT + ipilimumab 3mg/kg + nivolumab 1mg/kg q3wks x 4, then nivolumab 3mg/kg q2wks | PR at 17 weeks, ongoing at 8 mos. Pt died at 10 mos of complications related to encephalopathy | - | - | - | - | Case 2 described in manuscript |
| 3 | 59 M,  Positive | Multiple systemic therapies prior to anti PD-1 | Variable | Pembrolizumab + MCPyV-specific T cells | PD at 2 months & at 4 months | Ipilimumab 0.5mg/kg initially (Pembrolizumab added later) | Near CR lasting 2 years | Multiple systemic therapies after Ipilimumab | PD | Case 3 described in manuscript |
| 4 | 71 M, Positive | Multiple systemic therapies prior to anti PD-1 | Variable | Nivolumab | CR lasting 26 months, then PD | Ipilimumab 1mg/kg + Nivolumab Q6Wks ongoing | CR lasted 10 months | - | - | Case 4 described in manuscript |
| 5 | 64 F, unknown | Pembrolizumab 2mg/kg q3wks | PD at 4 mos | Ipilimumab 3mg/kg IV every 3 weeks | Died at 10 weeks from PD | - | - | - | - | Immune-mediated colitis after 2 doses of ipilimumab |
| 6 | 51 M,  Unknown | Pembrolizumab 2mg/kg q3wks | CR for 14 mos, then PD in CNS only | RT + ipilimumab 3mg/kg + nivolumab 1mg/kg q3wks x 4 | PD; died at 6 months from leptomeningeal MCC | - | - | - | - | PD in CNS only |
| 7 | 67 F,  Unknown | RT + Pembrolizumab 2mg/kg q3wks | PD at 2 months | RT + ipilimumab 1mg/kg x 1 | Ipilimumab discontinued due to toxicity; PD at 3 months | RT+ avelumab 10 mg/kg q2wks | PD at 2 months | - | - | History of CLL |
| 8 | 75 M,  Unknown | Avelumab 10 mg/kg q2wks | PD at 16 wks | Nivolumab 3mg/kg q3wks + Ipilimumab 1mg/kg q6wks | PD at ~9 wks | RT | Partial regression of irradiated lesions | - | - | - |
| 9 | 21 F, Positive | Nivolumab, Avelumab | PD | Avelumab + IFN + MCPyV-specific T cells | PD at 1 month | Ipilimumab 1mg/kg + Nivolumab x1 dose | PD | Multiple systemic therapies | PD | - |
| 10 | 71 M, Negative | Pembrolizumab | PR lasting 6 months | Ipilimumab 0.5mg/kg + Pembrolizumab x4 doses | PD at 3 months | Ipilimumab 1mg/kg + Pembrolizumab | PD at 7 months | - | - | Developed colitis after ipilimumab 1mg/kg |
| 11 | 63 M, Positive | Avelumab +RT+ MCPyV-specific T cells | PD | Ipilimumab 1mg/kg + Nivolumab | PD at 3 months | Multiple systemic therapies | PD | - | - | - |
| 12 | 67 M, Negative | Avelumab | PR lasting 12 months | Ipilimumab 1mg/kg + Nivolumab x4 doses | Stable disease for 3 months | Nivolumab | PD at 1 month | - | - | - |
| 13 | 63 M, Negative | Adjuvant Avelumab | PD at 2 months | Ipilimumab 1mg/kg + Nivolumab x2 doses | PD | - | - | - | - | - |

Table S1: Therapies administered and corresponding disease outcomes for patients with advanced Merkel cell carcinoma refractory to anti-PD-1 or anti-PD-L1. (CLL, chronic lymphocytic leukemia; CR, complete response; PR, partial response; irPR, immune-related partial response; MCPyV, Merkel cell polyomavirus; PD, progressive disease; RT, radiotherapy)
